# Impacts of dietary supplementation with nano-iron and methionine on growth, blood chemistry, liver biomarkers, and tissue histology of heat-stressed broiler chickens

**DOI:** 10.1007/s11250-022-03130-w

**Published:** 2022-03-05

**Authors:** Haidy G. Abdel-Rahman, Heba A. Alian, Manal M. A. Mahmoud

**Affiliations:** 1 Department of Clinical Pathology, Faculty of Veterinary Medicine, Suez Canal University, Ismailia, 41522 Egypt; 2 Department of Nutrition and Clinical Nutrition, Faculty of Veterinary Medicine, Suez Canal University, Ismailia, 41522 Egypt

**Keywords:** Broilers, Growth performance, Nano-iron, Feed additives, Hematology, Thermal stress

## Abstract

A 28-day study was done to explore the impact of nano-iron alone or combined with methionine on growth, blood chemistry, liver biomarkers, and tissue histology of heat-stressed chicken. One-day-old Ross 308 chicks were randomly allocated to three groups. Each group was divided into three replicates (13 chicks/replicate). The first group was the control one that was fed a basal diet without supplementation (T0). The second group was fed a basal diet with nano-iron 4 mg kg^−1^ diet (T1). The third group was fed a basal diet with nano-iron 4 mg kg^−1^ diet plus methionine 4 g kg^−1^ diet (T2). The results showed that the birds in the control group had significantly (*p* < 0.05) higher final weights. Also, a partial relief of heat stress adverse effects was observed on growth by T1 compared to T2. The T2 showed a significantly increased (*p* < 0.05) free iron (Fe) level and transferrin saturation index. Likewise, T2 significantly (*p* < 0.05) reduced total iron-binding capacity (TIBC) and transferrin level in comparison with T0 and T1. Also, hepatic impairment and inflammatory response were observed in the T2 group when compared to T0 and T1, besides a bad lipid profile. Further, T2 showed raised levels of Fe and ferritin in their hepatic tissues compared to those T1 and T0. A significant increment of thiobarbituric acid reactive and decrement of reduced glutathione levels in the hepatic tissues of T2 and T1 versus T0 levels were recorded. It is concluded that nano-iron at the level of 4 mg kg^−1^ in this study is highly absorbed, leading to harmful effects. Further investigations are needed to detect the proper supplemental level.

## Introduction

Heat stress is a vital environmental stressor challenging poultry production. It may significantly affect poultry performance, especially if coupled with high humidity, causing poultry suffering and may lead to death (Laganá et al. 2007). To deal with heat stress problems, researchers studied environment management as housing design, ventilation, etc. Another approach was nutritional management like ration formulation, adding feed additives, and supplementing water with electrolytes. The efficiency of some of these solutions was inconsistent (Lara and Rostagno 2013). Nano-technology is an evolving technology that has great potentials and various applications in animal nutrition. Nano-technology uses matter with 1 to 100 nm with new different characteristics. Trace minerals are introduced in small amounts in poultry nutrition. Trace minerals efficacy is limited by bioavailability, antagonism, and excretion rate from the body. The bioavailability of nanoparticles can be increased as they have different physical and chemical properties than their original corresponding mineral (Raje et al. 2018). The nanoparticle has a lower antagonism in the intestine that leads to improved absorption, reduced excretion to the environment, and improved feed efficiency (Gopi et al. 2017).

Iron (Fe) is an essential mineral routinely supplemented with broilers’ diet. It is an essential part of numerous enzymes and proteins that have a role in oxygen transport, maintaining health, and regulates cell growth and differentiation (Hänsch and Mendel 2009). Additionally, it is essential in the hemoglobin, myoglobin, and RBCs synthesis (Underwood 1999). By assisting enzymes, it has a major role in the tricarboxylic acid cycle facilitates the removal of harmful metabolites through Fe-containing peroxidases and catalases (Nikonov et al. 2011). Heat stress causes a decrease in Fe concentrations in serum and tissue (Combs and Combs 1984). Fe reduction leads to a collapse of the immune and antioxidant system with severe effects on birds’ health (Sahin et al. 2001). Methionine (Met) is a limiting amino acid in the poultry diet. It has a significant role in protein metabolism (a substantial methyl group donor), feather development, and immune system (Lai et al. 2018). Also, methionine is an essential factor in heat stress resistance. It is a glutathione precursor; it relives reactive oxygen species (ROS); therefore, supplementation of methionine can contribute to the antioxidant status of poultry (Sahebi Ala et al. 2019). Methionine can be gained through diet; therefore, much or less Met consumption can result in DNA methylation shift (Niculescu and Zeisel 2002), which may, in turn, support genomic insecurity (Arai and Kanai 2010). Hence, the purpose of this study was to evaluate the impact of dietary supplementation with nano-iron alone or combined with methionine on growth performance, physiological parameters of blood, and the microstructure of selected internal organs in heat-stressed chickens that kept at 35 ± 2 °C.

## Materials and methods

### Experimental birds and management

One-day-old Ross broiler chicks (*n* = 117) were obtained from a commercial company. Chicks were raised in a controlled brooder house in a deep litter system; each replicate pen was a 1-m^2^ area (100 × 100 × 100 cm). Chicks had ad libitum access to feed and water. The temperature was kept at 35 ± 2 °C with 65% relative humidity along the experimental period. The lighting pattern in the first week was 23-h light:1-h dark and then 2 h of darkness and 22-h light up to the end of the experiment. For food, water, and mortality, the chicks were checked three times daily (at 6:00 am, 2:00 pm, and 10:00 pm). Chicks were vaccinated according to the routine vaccination program and managed according to Ross broilers management handbook (Aviagen 2014a). The experiment was done in compliance with the guidelines of the Scientific Research Ethics Committee, Faculty of Veterinary Medicine, Suez Canal University, Egypt (Approval No. 2020043).

### Nano-Fe preparation

**Fig. 1:**
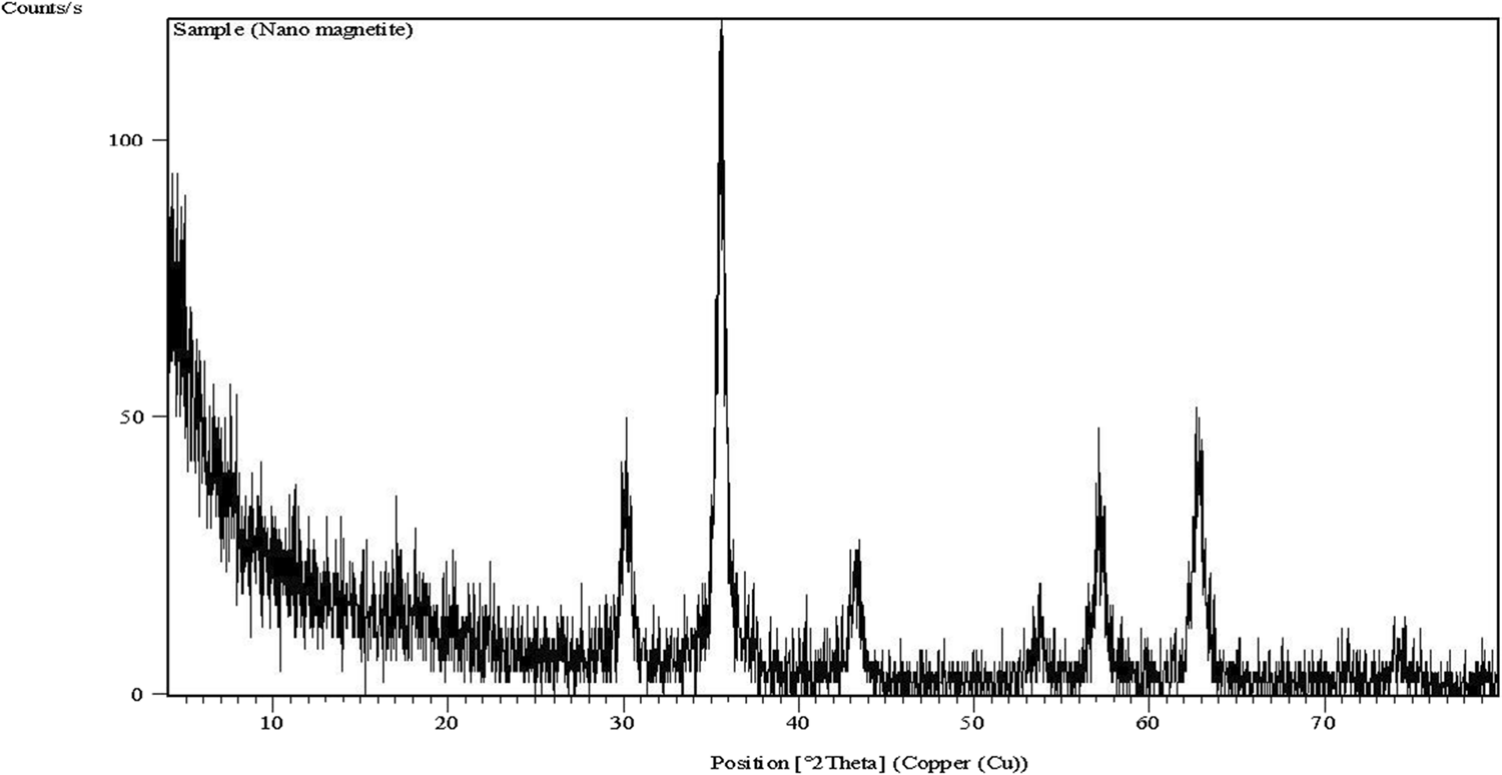
Characterization of magnetite nanoparticles by XRD (X-ray diffraction) techniques

**Fig. 2:**
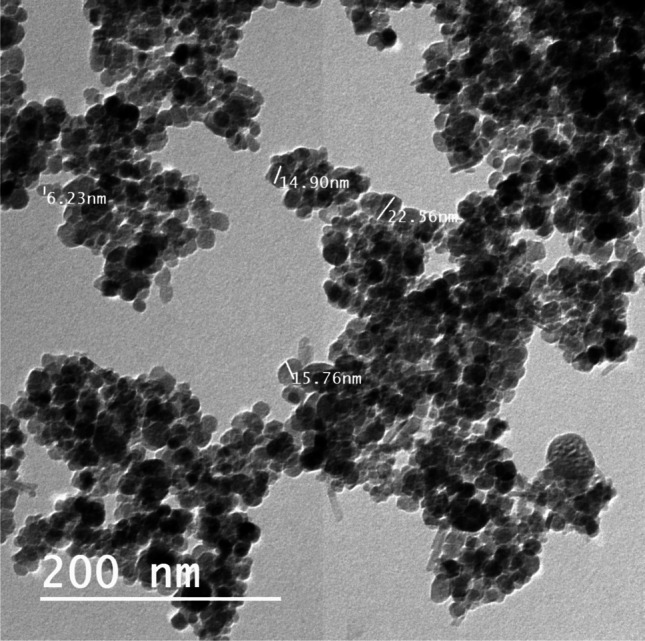
Characterization of magnetite nanoparticles by TEM (transmission electron microscopy) techniques (average 12.22 nm)

At room temperature, magnetite nanoparticles have been synthesized using a simple reverse co-precipitation method. Ferrous sulfate powder was used as an iron precursor, and ammonium hydroxide as a precipitating agent according to Mahmed et al. (2011). These particles were characterized by X-ray diffraction (XRD) (Fig. 1) and transmission electron microscope (TEM) (Fig. 2).

### Experimental design

Chicks were randomly assigned to three experimental groups. Each group was allocated to three replicates (13 chicks/replicate). The first group was the control one fed the basal diet without supplementation (T0). The second group was fed a basal diet supplemented with nano-iron 4 mg kg^−1^ diet. The third group was fed a basal diet supplemented with nano-iron 4 mg kg^−1^ diet plus methionine 4 g kg^−1^ diet. The basal diets were prepared according to the nutrient specification of Ross (Aviagen 2014b). Chicks were fed the diets for 28 days. The nutrient composition of the feed ingredients was carried out by a proximate chemical analysis method (AOAC 1990) (Table 1). Each complete feed has been analyzed and the data given (Table 2).

**Table 1:** Experimental diets composition as fed basis^a^

Ingredients%	Stater (0–10 day)	Grower (11–28 day)
Ground yellow corn (8% CP)^b^	55.51	58.73
Soya bean meal (46% CP)^b^	32.52	28.70
Corn gluten (60% CP)^b^	6.00	6.00
Soybean oil	1.55	2.615
Di-calcium-phosphate (22% Ca and 19% P)	2.00	1.77
Limestone (38% Ca)	1.38	1.23
DL-methionine (purity 99%)	0.28	0.23
L-Lysine (purity 99%)	0.16	0.125
Iodized sodium chloride	0.30	0.30
Vitamin premix^c^	0.15	0.15
Mineral premix^c^	0.15	0.15
Total	100.0	100.0
Calculated composition		
Crude protein (%)	23.0	21.5
ME (kcal per kg)	3000.0	3100.0
Calorie/protein ratio (C/P)	130.43	144.18
Calcium (%)	0.96	0.87
Available phosphorus (%)	0.3	0.435
Methionine (%)	0.22	0.27
Iron (%)	0.888	0.852

^a^Formulated according to Ross nutrition specifications (2017) (Ross 2017)

^b^Chemical analysis was performed according to AOAC (AOAC 1990)

^c^Each 3 kg of vitamin and mineral premix contains the following: vit. A 12 mIU, vit. D_3_ 3 mIU, vit. E 40,000 mg, vit. k_3_ 4000 mg, vit. B_1_ 4000 mg, vit. B_2_ 15,000 mg, vit. B_6_ 5000 mg, vit. B_12_ 30 mg, biotin 300 mg, pantothenic acid 20,000 mg, nicotinic acid 60,000 mg, folic acid 3000 mg, magnesium sulfate 1,200,000 mg, zinc sulfate 100,000 mg, iron sulfate 80,000 mg, copper sulfate 30,000 mg, potassium iodide 3000 mg, sodium selenate 200 mg, cobalt sulfate 100 mg, carrier (CaCO_3_) to 3 kg (High mix premix—Alpha Pharm El Asher, Egypt. Patch No. 614241, production 2–2017)

**Table 2:** Chemical analysis of the basal diet on DM basis

Chemical composition^a^	Starter (0–10 day) diet	Grower (11–28 day) diet
Moisture	6.83	6.70
Crude protein (CP)	23.12	21.30
Ether extract (EE)	3.75	3.49
Ash	6.11	6.43
Crude fiber (CF)	3.52	3.75
NFE^b^	56.67	58.33
Total	100	100

^a^Chemical analysis was performed according to AOAC (AOAC 1990)

^b^NFE = 100 − (moisture% + CP% + EE% + Ash% + CF%)

### Growth performance parameters

Performance parameters were done according to Chen et al. (2013). Weekly measurements were recorded using a sensitive balance (0.0001 g, Fisher Scientific) of individual body weight and feed consumption/replicate. Body weight gains (BWG) and feed conversion ratio (FCR) were calculated accordingly. Mortality and chick’s health were observed daily, and mortality percentage was calculated. After 28 days of study, two birds from each replicate were randomly chosen to be slaughtered by manual slaughtering using a sharp knife. Birds were de-feathered, eviscerated, and then weighed. Internal organs (gizzard, heart, and liver) were weighed and expressed as a percentage of body weight (Selim et al. 2015).

### Sampling

On the 28th day of the feeding trial, two birds/replicate were selected and fasted for feed only overnight, then were slaughtered. Blood samples were collected with and without anticoagulants. Blood samples were centrifuged at 3000 rpm for 15 min, and serum was preserved at − 20 °C until performing the biochemical analysis. Tissue samples (liver, heart, intestine, and spleen) of the same slaughtered birds were collected handled for histopathological assessment. A part from the liver samples was taken and kept at − 20 °C for performing some chemical analysis in liver tissue.

### Hematological examination

At the end of the experiment, blood samples were collected from the two birds per replicate for hematological analysis. Erythrogram and total leukocytic count were determined in whole blood samples of chickens in different experimental groups following standard methods as described by Campbell and Ellis (2013). The hematological analysis includes red blood cells count (RBCs), hemoglobin (Hb), packed cell volume (PCV), mean corpuscular volume (MCV), mean corpuscular hemoglobin (MCH), mean corpuscular hemoglobin concentration (MCHC), and total leukocytic count (TLC).

### Serum biochemical assessment

Iron concentration (Fe), total iron-binding capacity (TIBC), total protein (TP), albumin, alanine aminotransferase (ALT), total cholesterol (CHO), triglycerides (TGs), and high-density lipoprotein cholesterol (HDL-C) concentration were determined in serum using kits of CliniChem Ltd., Budapest, Hungary. Globulin was calculated as the difference between total protein and albumin (Kaneko et al. 2008). Low-density lipoprotein cholesterol (LDL-C) was calculated according to Friedewald et al. (1972) formula described by Davidson and Rosenson (2009). LDL cholesterol (mg/dL) = total cholesterol − (TGs/5 + HDL-C).

Serum transferrin was estimated using ELISA kit manufactured by Wuhan Fine Biological Technology Co., Ltd., Wuhan, Hubei, China. Serum tumor necrosis factor (TNF-α) was assessed by ELISA kit of CUSABIO, Houston, TX. Transferrin saturation index was estimated according to the following equation (Bain et al. 2017): Transferrin saturation index (%) = (serum iron × 100)/TIBC.

The hepatic ferritin content was recorded by ELISA kit of LifeSpan BioSciences, Inc., Seattle, WA, USA. Chicken reduced glutathione was estimated using an enzyme-linked immunosorbent assay (ELISA) kit (MYBIOSOURCE (MBS), San Diego, USA). The thiobarbituric acid reactive (TBARS) assay in hepatic tissue homogenate was determined by CELL BIOLABS Inc., San Diego, USA.

### Histopathological examination

Specimens from the liver, heart, intestine, and spleen of slaughtered birds were fixed in 10% formalin, pH = 7.2. The fixed tissues were routinely embedded in paraffin, cut into 5 μm thick sections, and processed for hematoxylin and eosin staining, according to Bancroft and Gamble (2008). Morphometric examination of the tissue sections was done using a computer-assisted microscopic image analysis system.

#### Tissue homogenate preparation

A part from liver tissues was collected and homogenized in PBS (pH = 7.4) in the ratio of 1:10 in a glass homogenizer. The homogenate was centrifuged, and the supernatant was taken for later biochemical assays by spectrophotometer.

### Statistical analysis

Collected data from all groups were statistically analyzed to compare means with the control group using a statistical software program (IBM SPSS for Windows, version 22, USA). Differences among means of the experimental groups were evaluated using one-way analysis of variance (ANOVA) with Duncan tests, according to Chambers et al. (2017). A *p* value < 0.05 was used to display the significance between all groups.

## Results

The final weight (614.17 g/bird/day) and weight gain (20.43 g/bird/day) were significantly (*p* < 0.05) improved in T1 (nano-Fe) compared to T2 (nano-Fe plus methionine), but significantly lower than the control. Feed intake was not significantly (*p* < 0.05) different among all groups. FCR was not significantly different between the control and T1. FCR was significantly improved in T1 compared to T2. Mortality was not significantly different among all groups. Gizzard percentage and liver percentage were not significantly different among all groups. Heart percentage (20.43%) was significantly higher in T2 compared to the control (Table 3). Moreover, the hematological examination of blood samples collected from all groups did not reveal significant changes among experimental groups (Table 4).

**Table 3:** Effect of experimental diets on broilers performance at 28th day

Parameters	Groups		
	Control (T0)	Nano-Fe (T1)	Nano-Fe plus methionine (T2)
Initial weight (g/bird)	46.73 ± 0.61^a^	47.12 ± 1.14^a^	46.38 ± 1.30^a^
Final weight (g/bird)	774.63 ± 17.61^a^	614.17 ± 18.17^b^	524.21 ± 19.08^c^
Feed intake (g/bird/day)	39.10 ± 0.82^a^	36.78 ± 0.78^a^	36.24 ± 1.06^a^
Weight gain (g/bird/day)	26.01 ± 0.33^a^	20.43 ± 0.18^b^	17.07 ± 0.72^c^
FCR (g:g)	1.51 ± 0.01^b^	1.76 ± 0.04^b^	2.16 ± 0.12^a^
Mortality %	7.69 ± 0.00^a^	12.82 ± 2.56^a^	17.95 ± 5.13^a^
Internal organs weight %			
Gizzard	2.29 ± 0.15^a^	2.79 ± 0.16^a^	2.73 ± 0.22^a^
Heart	0.59 ± 0.03^b^	0.73 ± 0.07^ab^	0.78 ± 0.06^a^
Liver	3.01 ± 0.22^a^	3.13 ± 0.17^a^	3.54 ± 0.16^a^

^a–^^c^Means in the same row with different superscripts are significantly different (*p* < 0.05); values are presented as means ± SEM

**Table 4:** Effect of experimental diets on hematological examination of broilers at 28th day

Parameters	Groups		
	Control (T0)	Nano-Fe (T1)	Nano-Fe plus methionine (T2)
RBCs (10^6^ μL^−1^)	2.33 ± 0.12^a^	2.57 ± 0.13^a^	2.51 ± 0.19^a^
Hb (g dL^−1^)	9.27 ± 1.12^a^	11.51 ± 0.87^a^	10.50 ± 0.27^a^
PCV (%)	27.00 ± 0.91^a^	29.50 ± 3.60^a^	29.25 ± 1.25^a^
MCV(fl)	116.90 ± 7.44^a^	115.06 ± 13.40^a^	117.94 ± 7.00^a^
MCH (pg)	40.42 ± 6.38^a^	45.33 ± 5.09^a^	42.60 ± 3.31^a^
MCHC (g dL^−1^)	34.07 ± 3.08^a^	41.37 ± 6.58^a^	36.14 ± 2.03^a^
TLC (10^3^ μL^−1^)	14.75 ± 1.70^a^	17.50 ± 1.55^a^	17.75 ± 2.63^a^

Values are presented as means ± SEM

*RBCs* red blood cells count, *Hb* hemoglobin, *PCV* packed cell volume, *MCV* mean corpuscular volume, *MCH* mean corpuscular hemoglobin, *MCHC* mean corpuscular hemoglobin concentration, *TLC* total leukocytic count

As for the serum biochemical investigations, T2 and T1 chicken showed significantly (*p* < 0.05) increased free iron (Fe) level and transferrin saturation index compared to T0. Likewise, T2 and T1 significantly (*p* < 0.05) diminished total iron-binding capacity (TIBC) and transferrin level in comparison with T0. Furthermore, T2 differed significantly in the investigations mentioned above when compared to T1 (Table 5).

**Table 5:** Effect of experimental diets on serum iron, TIBC, and transferring concentrations of broilers at 28th day

Parameters	Groups		
	Control (T0)	Nano-Fe (T1)	Nano-Fe plus methionine (T2)
Fe (μg dL^−1^)	132.97 ± 0.69^c^	152.90 ± 2.11^b^	171.50 ± 2.09^a^
TIBC (μg dL^−1^)	337.73 ± 3.12^a^	303.50 ± 3.32^b^	282.50 ± 1.95^c^
Transferrin (ng mL^−1^)	258.23 ± 4.99^a^	228.23 ± 2.47^b^	208.07 ± 1.35^c^
Trans. Sat. index (%)	39.77 ± 0.29^c^	50.37 ± 0.16^b^	60.72 ± 1.13^a^

^a–^^c^Means in the same row with different superscripts are significantly different (*p* < 0.05); values are presented as means ± SEM

*Fe* iron (Fe), *TBIC* total iron-binding capacity, *Trans. Sat. index* transferrin saturation index

Also, hepatic impairment and inflammatory response were observed in the T2 group when compared to T0 and T1 groups; evidenced by a significant elevation in ALT activity (33.85 UL^−1^) and TNF-α level (13.16 pg mL^−1^), together with significant (*p* < 0.05) decrement in TP, albumin, and globulin concentrations in addition to bad lipid profile manifested by raised CHO, TGs, and LDL-C values with a reduced HDL-C values (Tables 6 and 7).

**Table 6:** Effect of experimental diets on serum proteinogram, ALT activity, and TNF-α level of broilers at 28th day

Parameters	Groups		
	Control (T0)	Nano-Fe (T1)	Nano-Fe plus methionine (T2)
TP (g dL^−1^)	4.83 ± 0.03^a^	4.53 ± 0.03^b^	4.15 ± 0.04^c^
Albumin (g dL^−1^)	2.57 ± 0.05^a^	2.28 ± 0.02^b^	1.95 ± 0.04^c^
Globulin (g dL^−1^)	2.26 ± 0.02^a^	2.25 ± 0.01^a^	2.20 ± 0.01^b^
ALT (UL^−1^)	19.94 ± 0.16^c^	25.73 ± 0.49^b^	33.85 ± 1.26^a^
TNF-α (pg mL^−1^)	7.62 ± 0.02^c^	10.28 ± 0.24^b^	13.16 ± 0.16^a^

^a–^^c^Means in the same row with different superscripts are significantly different (*p* < 0.05); values are presented as means ± SEM

*TP* total proteins, *ALT* alanine aminotransferase, *TNF-α* tumor necrosis factor alpha

**Table 7:** Effect of experimental diets on serum lipid profile of broilers at 28th day

Parameters	Groups		
	Control (T0)	Nano-Fe (T1)	Nano-Fe plus methionine (T2)
CHO (mg dL^−1^)	198.53 ± 2.14^c^	220.97 ± 2.11^b^	242.50 ± 3.03^a^
TGs (mg dL^−1^)	111.17 ± 2.39^c^	127.37 ± 2.00^b^	144.83 ± 1.61^a^
LDL-C (mg dL^−1^)	129.90 ± 2.14^c^	153.16 ± 1.40^b^	174.87 ± 3.00^a^
HDL-C (mg dL^−1^)	46.40 ± 0.62^a^	42.33 ± 0.35^b^	38.67 ± 0.38^c^

^a–^^c^Means in the same row with different superscripts are significantly different (*p* < 0.05); values are presented as means ± SEM

*CHO* cholesterol, *TGs* triglycerides, *LDL-C* low-density lipoprotein cholesterol, *HDL-C* high-density lipoprotein cholesterol

Oxidative state assessment of the hepatic tissues harvested from experimental chicken revealed significant (*p* < 0.05) increment of TBARs and decrement of GSH levels of T2 and T1 versus T0 levels. Moreover, T1 manifested relieved lipid peroxidation and increased GSH level (171.10 mg g^−1^) significantly (*p* < 0.05) when compared to the T2 group. Over and above, T2 showed significantly (*p* < 0.05) raised levels of Fe (380.37 μg g^−1^) and ferritin (253.33 μg g^−1^) in their hepatic tissues when compared to those levels of T1 and T0 groups (Table 8).

**Table 8:** Effect of experimental diets on hepatic tissue concentration of Fe and ferritin and oxidative stress indices of broilers at 28th day

Parameters	Groups		
	Control (T0)	Nano-Fe (T1)	Nano-Fe plus methionine (T2)
Fe (μg g^−1^)	328.90 ± 1.36^c^	355.00 ± 2.08^b^	380.37 ± 4.85^a^
Ferritin (μg g^−1^)	216.03 ± 1.39^c^	239.10 ± 1.36^b^	253.33 ± 2.34^a^
GSH (mg g^−1^)	171.10 ± 1.85^a^	152.43 ± 1.68^b^	125.83 ± 1.80^c^
TBARS (nmol g^−1^)	15.09 ± 0.36^c^	21.35 ± 0.49^b^	27.04 ± 0.65^a^

^a–^^c^Means in the same row with different superscripts are significantly different (*p* < 0.05); values are presented as means ± SEM

*Fe* iron, *GSH* reduced glutathione, *TBARs* thiobarbituric acid reactive

**Fig. 3:**
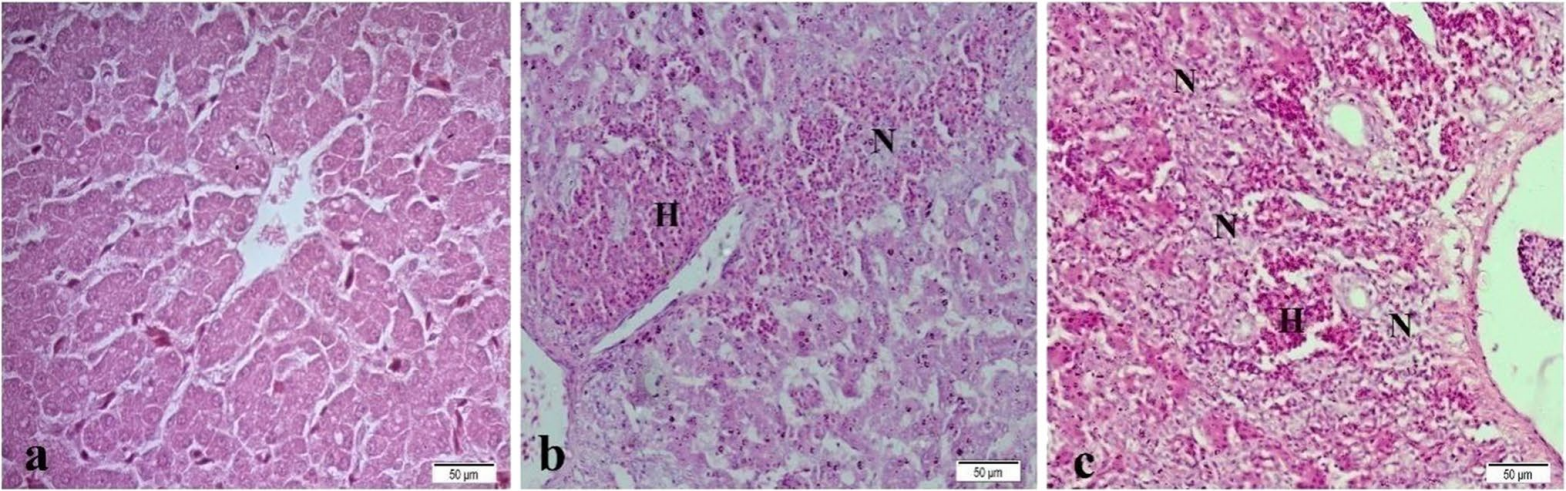
Liver showing normal intact hepatic cords and hepatocytes in control group (**a**), mild focal hemorrhage (H) and discrete necrosis (N) of some hepatocytes in nano-Fe group (**b**), and multifocal hemorrhage and necrosis in nano-Fe + Met group (**c**). H&E, × 40

**Fig. 4:**
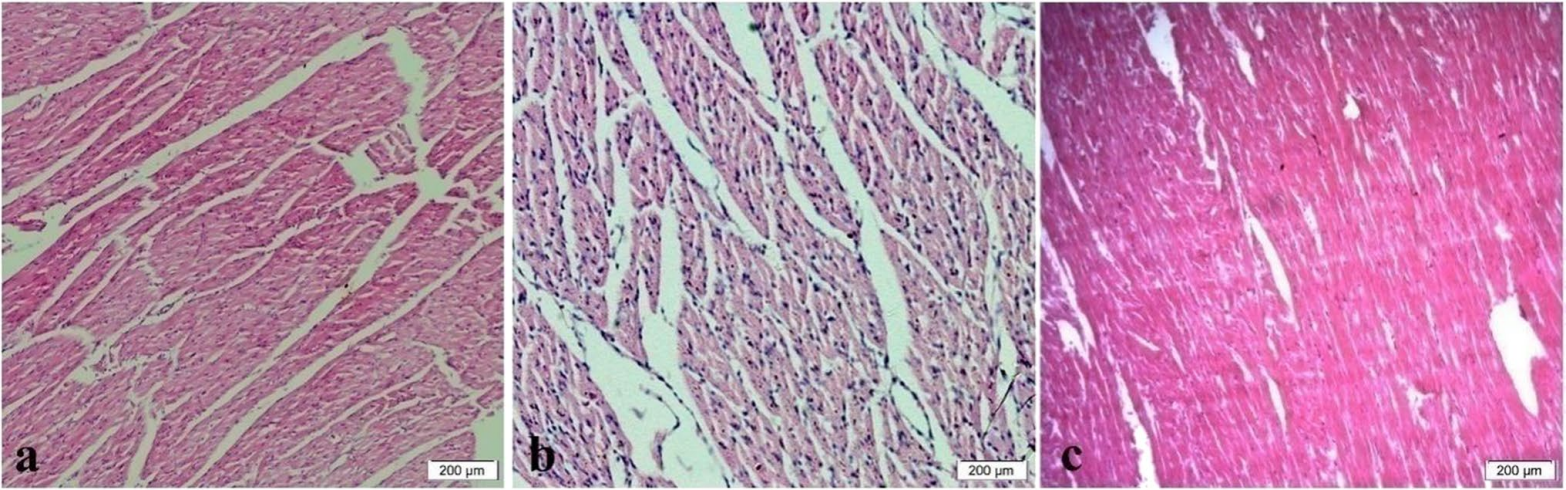
Heart showing normal heart muscles in control group (**a**), mild hypertrophy and edema in nano-Fe group (**b**), and mild to moderate hypertrophy in nano-Fe + Met group (**c**). H&E, × 200

**Fig. 5:**
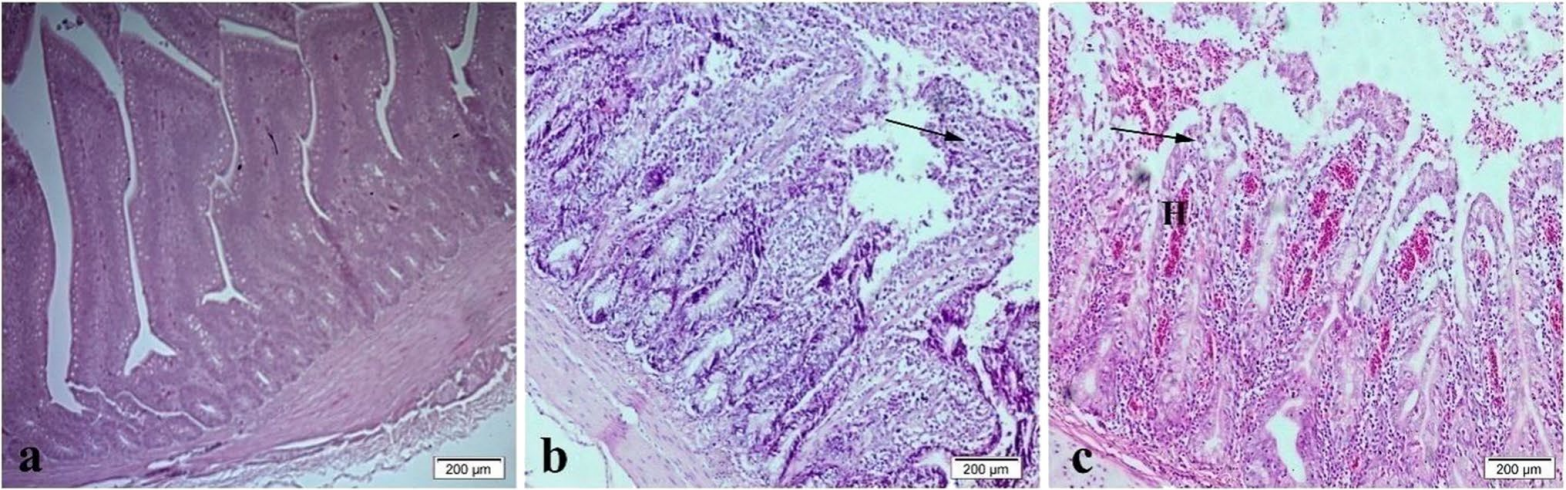
Intestine showing normal intact intestinal villi in control group (**a**), mild hyperplasia of the tips of intestinal villi (arrow) along with mild mucinous degeneration in nano-Fe group (**b**), and mild focal hemorrhage (H) in nano-Fe + Met group (**c**). H&E, × 200

**Fig. 6:**
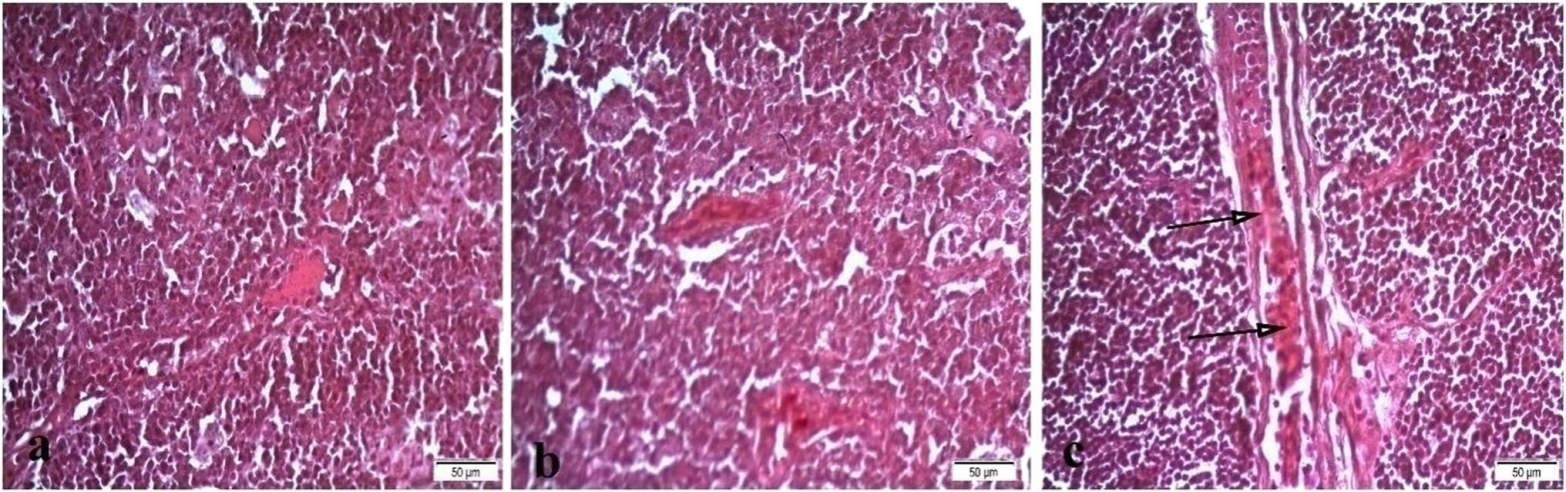
Spleen showing normal splenic white and red pulps in both control group (**a**) and nano-Fe group (**b**) and moderate congestion of blood vessels and sinusoids in nano-Fe + Met group (**c**). H&E, × 40

The histopathological examination of the liver tissue showed normal intact hepatic cords and hepatocytes in T0 (Fig. 3a), mild focal hemorrhage and discrete necrosis of some hepatocytes in T1 (Fig. 3b), and multifocal hemorrhage and necrosis in T2 (Fig. 3c). Heart revealed normal heart muscles in T0 (Fig. 4a), mild hypertrophy and edema in T1 (Fig. 4b), and mild to moderate hypertrophy in T2 (Fig. 4c). Intestine showed normal intact intestinal villi in T0 (Fig. 5a), mild hyperplasia of the tips of intestinal villi along with mild mucinous degeneration in T1 (Fig. 5b), and mild focal hemorrhage in T2 (Fig. 5c). Spleen showed normal splenic white and red pulps in both T0 and T1 (Fig. 6a, b) and moderate blood vessels and sinusoids congestion in T2 (Fig. 6c).

## Discussion

Poultry impaired growth performance due to heat stress has been observed in many studies (Attia et al. 2011; Ghazi et al. 2012; Imik et al. 2012; Niu et al. 2009). The effectiveness of most published variable interventions has been inconsistent. In addition, the fundamental mechanisms associated with these effects, and those related to broiler behavior and welfare, are scarce (Lara and Rostagno 2013).

The results indicated that the birds in the control group had significantly higher final weights. Also, a partial relief of heat stress adverse effects was observed on growth by T1 compared to T2. This agrees with Harper et al. (1970) who observed a retarded rat growth and a decreased feed intake upon excess supplementation with methionine. Li et al. (2020) stated that consuming diets with low or excessive methionine levels leads to adverse effects on growth performance. In addition, the negative impact may be due to the methionine source as it was used DL-methionine. Willemsen et al. (2011) concluded that the synthetic analog 2-hydroxy-4-methylthiobutanoic acid (DL-HMTBA) supplementation partially relived the growth performance effects of chronic heat stress compared to DL-methionine supplementation.

Additionally, a significant decrease of albumin concentration in T1 and T2 was observed in comparison to T0. This may explain the reduced final body weight in both groups. As albumin synthesized mainly in the liver is the major source for amino acid formation. Protein synthesis is decreased due to liver malfunction, resulting in less muscles formation, which gives rise to decreased final body weight.

In normal regular rearing conditions (without heat stress), nano-Fe supplemented in broiler diets improved body weight with no effect on the composition of liver, thigh, and breast (Sizova et al. 2016). Supplementing nano-Fe in combination with arginine, lysine, and methionine enhanced iron utilization efficiency and growth in chicken (Rahmatollah et al. 2018).

Studies using nano-Fe as a dietary supplement to poultry under heat stress conditions are rare. Ramiah et al. (2019) observed that zinc oxide nanoparticles relieved the adverse effects of heat stress in poultry. They recommended further studies to determine the optimal nano-Zn supplementation level. El-Kassas et al. (2018) showed that nano-Cu might be used under normal regular rearing temperature to enhance immunity and during heat stress to lower its effects depending on the magnitude of the heat stress. Hajializadeh et al. (2017) indicated that nano-chromium improved weight gain and FCR of heat-stressed broilers. Also, nano-Se supplementation alleviated adverse effects of heat stress on poultry performance (Mahmoud et al. 2016). The disagreement of this study with the other studies may be due to using nano-Fe, method of nano-Fe preparation, the applied level of nano-Fe and methionine, the chronic high ambient temperature the chicks were subjected to, the diet composition, and the size of the nanoparticle. The general rule is the smaller the nanoparticles are, the more they are absorbed and the deeper they are transported and goes into the body systems. The smaller particles taken up by the villus epithelium directly entered the bloodstream and were distributed all over the body (Thulasi et al. 2013).

Mortality was not significantly different among all groups; however, there was a trend towards lower mortality in the control group. This is in accordance with Hassan et al. (2007), who observed a non-significant increase in mortality rate in heat-stressed chicks compared to the control and the mortality rates were close to the control by the end of the experiment. This may be due to the adaptation of birds to heat stress (Hassan et al. 2007).

Cytotoxicity can evolve due to iron oxide nanoparticles (IONPs) administration through function impairment of different cellular organelles: mitochondria, nucleus, and DNA (Oberdörster et al. 2007). Moreover, Gaharwar et al. (2017) recorded increased redox production via reduced SOD, CAT, and GSH concentrations with increased LPO and DNA strand break, decreased cell viability, and increased lactate dehydrogenase release in rat’s splenic lymphocytes, thus proving that IONPs can result in cellular toxicity and damage. In an in vivo study on male Wistar rats, IONPs treatment revealed a dose-dependent reduction in hepatic tissue GSH level, SOD, and CAT activities together with excess in MDA level in the hepatic tissue of rats (Ansari et al. 2019). Lipid peroxidation can lead to deformity in the cell membrane structure and shift in its permeability, thus hindering various metabolic operations (Nigam and Schewe 2000). Moslemi et al. (2013) reported increased hepatic oxidative fatigue and atherosclerosis incidence in rats receiving nano-iron oxide. Overall, Sulaiman et al. (2015) recorded raised total cholesterol, triglycerides, and LDL-C, with diminished HDL-C concentration in serum of rats receiving oral administration of silver nanoparticles.

Reactive oxygen species generated, as a sequel of exposure to nanoparticles, can lead to cellular inflammation and apoptosis in lung and liver tissues (Sadeghi et al. 2015b), where they recorded increased free radicals production with diminished GSH level in lung tissue and also elevated AST, ALT, and ALP in the blood of adult male Wistar rats exposed to IONPs due to injury of hepatocytes. Furthermore, ROS enhancement stimulates TNF-α, interleukins, and kinases as signals of pro-inflammatory procedures (Li et al. 2010). A study stated that Kupffer cells and hepatocytes of adult female mice promptly picked gold nanoparticles (Sadauskas et al. 2007). Moreover, Parivar et al. (2016) declared that IONPs administration gave rise to iron accumulation in tissue, and though inducing toxicity. Not so far, Babadi et al. (2012) confirmed that IONPs accumulate in the hepatocytes of rats causing oxidative injury and elevated activities of serum liver enzymes (ALT, AST, and ALP).

Iron is fundamental for livestock; however, iron overload is virulent and fatal. It is well known that the liver is the main store for iron and is the most liable tissue for injury in case of iron accumulation due to iron overload (Bonkovsky 1991). Moreover, the liver has a part in the metabolism of sulfur-containing amino acids such as methionine (Toborek et al. 1996). Minerals chelation with amino acids facilitates their absorbance from the gut and increases their bioavailability in the body than supplemented as inorganic salts (oxides or sulfates). Indeed methionine is the usually used amino acid for chelation (Wedekind et al. 1992).

Our results of elevated hepatic concentrations of iron, ferritin, and TBARs in the T2 group were also previously recorded by Mori and Hirayama (2000) in rat’s liver when excess methionine was administered, where methionine was turned into a hepatotoxic agent affecting the iron metabolism and leading to accumulation of iron in the liver. Lynch and Strain (1989) suggested that increased methionine intake provoked oxidative metabolites generation from methionine metabolism, which resulted in diminished antioxidant parameters, iron accumulation, and lipid peroxidation via raised formation of free radicals resulting from autoxidation of methionine metabolites as homocysteine and cysteine (Toborek et al. 1996). Xie et al. (2004) conducted an experiment on Peking ducklings from hatch till 21 days of age with graded increased levels of methionine supplementation. They found that the increased methionine intake resulted in decreased weight gain and feed consumption by increased serum concentration of homocysteine. Mitchell and Lemme (2008) stated a higher absorption rate of methionine from the small intestine of heat-stressed broilers (35 °C) than normal temperature reared broilers (22 °C). Baggott and Tamura (2007) concluded that methionine supplemented with iron resulted in increased homocysteine concentrations, which induced oxidative stress and toxic effects on the cell. On the other hand, Miller et al. (2005) reported that mice supplemented with a Met-limited diet were resistant to oxidative injury of hepatocytes. One other thing, Donnik (2017) reported that feeding nano-Fe to poultry led to a raise in NO metabolites content in the liver and iron content in the body. A more significant increment was recorded with the extra feeding of amino acids. Additionally, Varkhede et al. (2019) investigated the effect of IONPs on rat growth hormone (rGH) oxidation under chemical stress and found that H_2_O_2_ oxidized the methionine residues of rGH and IONPs suppressed the oxidation when compared to phosphate buffer control. Also, Wu et al. (2014b) suggested that magnetic nanoparticles coated with amino acids induced toxicity of human cells expressed by cell apoptosis. Transferrin is synthesized by the liver (Fujii et al. 1996), so the low transferrin concentration may be attributed to liver disease, consequently decreasing the iron-binding capacity. The same, in body iron overload condition; the blood transferrin level decreases, thus TIBC decreases. In the case of hypoproteinemia, serum transferrin concentration is reduced (Evans et al. 2013). Park et al. (2007) stated that the excessive iron content in plasma than the iron-binding capacity of transferrin could accumulate of iron in the body, causing cell damage and inflammation.

These results can be confirmed from the histopathological examination of this study. It was declared that nano-iron supplemented group alone or combined with methionine was suffering from several pathological conditions. Certain liver, heart, intestine, and spleen lesions were reported in histopathological findings. A number of in vitro studies have associated with cytotoxicity induced by nano-iron with oxidative stress (Manke et al. 2013). Similarly, an increased generation of ROS by nano-iron exposure was previously observed (Sadeghi et al. 2015a; Wu et al. 2014a). Besides, nano-iron with methionine group showed several ultra-structural and histopathological lesions. Lynch and Strain (1989) suggested that high methionine intake provoked oxidative metabolites generation from methionine metabolism which resulted in reduced antioxidant status. Finally, ROS due to dietary nano-iron alone or combined with methionine are involved in the damage of organs architecture such as liver, heart, intestine, and spleen in our study outcomes. This is in accordance with that reported previously by Ramakrishnan et al. (2011) who revealed that iron induces toxicity to liver and kidney by means of oxidative stress by either free radicals over production or by impairing antioxidant abilities in broilers. Histopathology of bursa and spleen in iron toxic group (was fed 0.5% ferrous sulfate) revealed areas of necrosis and degenerative changes (Ramakrishnan et al. 2009). Besides, the intake of excess L-methionine by rats induces tissue damage including liver enlargement, fatty liver, and membrane damage of RBCs (Klavins et al. 1963).

In conclusion, an increase in iron intake above the NRC requirement may be beneficial. However, nano-iron (12.22 nm) at the level of 4 mg kg^−1^ in this study is highly absorbed, leading to harmful effects. Decreasing the used level needs more investigation to determine the proper level of nano-Fe supplementation in broilers.

## Data Availability

The datasets made and/or analyzed during this study are available from the corresponding author on reasonable request.
